# Vascular Hysteresis Loops and Vascular Architecture Mapping in Patients with Glioblastoma treated with Antiangiogenic Therapy

**DOI:** 10.1038/s41598-017-09048-w

**Published:** 2017-08-17

**Authors:** Andreas Stadlbauer, Max Zimmermann, Stefan Oberndorfer, Arnd Doerfler, Michael Buchfelder, Gertraud Heinz, Karl Roessler

**Affiliations:** 1 Department of Neurosurgery, University of Erlangen-Nürnberg, Erlangen, Germany; 2 Institute of Medical Radiology, University Clinic of St. Pölten, St. Pölten, Austria; 3 Department of Neurology, University Clinic of St. Pölten, St. Pölten, Austria; 4 Department of Neuroradiology, University of Erlangen-Nürnberg, Erlangen, Germany

## Abstract

In this study, we investigated the variability of vascular hysteresis loop (VHL) shapes and the spatial heterogeneity of neovascularization and microvascular alterations using vascular architecture mapping (VAM) in patients with recurrent glioblastoma during bevacizumab mono-therapy. VAM data were acquired in 13 patients suffering from recurrent glioblastoma prior to and 3 months after bevacizumab treatment onset using a dual contrast agent injections approach as part of routine MRI. Two patients were additionally examined after the first cycle of bevacizumab to check for early treatment response. VHLs were evaluated as biomarker maps of neovascularization activity: microvessel type indicator (MTI) and curvature (Curv) of the VHL-long-axis. Early response to bevacizumab was dominated by reduction of smaller microvasculature (around 10 µm). In the 3-month follow-up, responding tumors additionally showed a reduction in larger microvasculature (>20 µm). VAM biomarker images revealed spatially heterogeneous microvascular alterations during bevacizumab treatment. Responding, non-responding, progressive, and remote-progressive tumor areas were observed. MTI may be useful to predict responding and non-responding tumor regions, and Curv to assess severity of vasogenic edema. Analysis of VHLs in combination with VAM biomarkers may lead to a new perspective on investigating the spatial heterogeneity of neovascularization and microvascular alterations in glioblastoma during antiangiogenic therapy.

## Introduction

Glioblastoma (GB) is the most aggressive primary brain tumor in adults with an elevated expression of vascular endothelial growth factor (VEGF) protein, which has been identified as a critical regulator of tumor angiogenesis, endothelial cell proliferation, and migration^1^. Thereby, GBs are among the most vascularized of all solid tumors showing a disorganized, abundant and aberrant vasculature with tortuous vessels of variable diameter, heterogeneous distribution^2^, and abnormal capillary bed topology^3^. GBs are therefore attractive targets for antiangiogenic therapies (AAT)^4^ and the VEGF-specific antibody bevacizumab was approved by the Food and Drug Administration (FDA) for this purpose in 2009^5^. Administration of bevacizumab in humans has been shown that magnetic resonance imaging (MRI) based tumor volume criteria doesn’t make sense for response assessment in GBs^6^ even though its criteria have been updated by the Response Assessment in Neuro-Oncology (RANO) working group^7^. The major challenge is the decreased vessel permeability^8^, which results in diminished contrast agent extravasation^9^ but does not necessarily reflect biological tumor response^10^.

Computer simulations as well as experiments in animals and humans have demonstrated that more advanced MRI techniques can estimate microvascular vessel caliber, thereby providing further insight into tissue microvascularity^11–14^. The physical basis for MRI-based vessel caliber estimation is the different sensitivity of gradient-echo (GE) and spin-echo (SE) MRI to magnetic susceptibility^11–13^. The highly susceptibility-sensitive GE signals are dominated by a broad range of larger microvessel diameters (starting from 20 µm, i.e. larger arterioles and venules)^11^, with reduced sensitivity to the smaller microvascular range^15^. In contrast, despite its lower overall sensitivity to contrast agent-induced signal variations, SE signals exhibits a peak sensitivity to the microvasculature at a vessel diameter around 10 µm^11^, including capillaries and both small arterioles and venules. These differences in sensitivity to the vascular properties were shown in simulations studies^11, 14^ and verified in practice^16^.

Kiselev *et al*.^13^ were the first who reported about a “loop” in the parametric plot of the relaxation rates for GE (${\rm{\Delta }}{{\rm{R}}}_{2,\text{GE}}$) versus SE (${{\rm{\Delta }}{\rm{R}}}_{2,\text{SE}}^{3/2}$) first-pass peaks in dynamic susceptibility contrast (DSC) perfusion MRI. They found that the shape of the loop and the direction of its passage may differ between normal brain and tumor tissue. Xu *et al*.^17^ demonstrated by considering a tree model of microvasculature, that the direction of the loop is influenced mainly by the relative arterial and venous blood volume, as well as by the tracer bolus dispersion. These findings were confirmed by Hsu *et al*.^18^ by using a dual contrast agent injections approach. Emblem *et al*.^19^ recently detected an additional vasculature-dependent effect on the MR signals: a temporal shift between GE and SE signals, which influences the formation of the loop curve too. Depending on the hemodynamic properties of a tissue, the different sensitivities of the GE and SE signals to larger and smaller microscopic vessels resulted in a temporal shift between the respective MRI signal readouts. They demonstrated that patients with recurrent GBs, who responded to AAT with reduced tumor vessel calibers and improved hemodynamic efficiency survived longer than patients without these responses. This promising technique, however, has yet not found its way into clinical routine diagnostics. Reasons for this could be the lack of both clinically compatible MRI sequence protocols and imaging biomarkers considering the temporal shift phenomenon. Furthermore, a detailed analysis of the diversity of loop curve shapes in patients with glioblastoma and their changes during antiangiogenic therapy have not been presented so far.

In this study we used a MRI sequence protocol which is compatible to and practicable for clinical routine diagnostics in order to investigate the diversity and the changes of vascular hysteresis loop (VHL) shapes in patients suffering from recurrent GBs during bevacizumab mono-therapy. VHL analysis was extended with MRI biomarkers adapted to the temporal shift phenomenon. This approach, termed vascular architecture mapping (VAM), was used for investigating the spatial heterogeneity of neovascularization activity and microvascular changes due to bevacizumab treatment.

## Results

**Figure 1:**
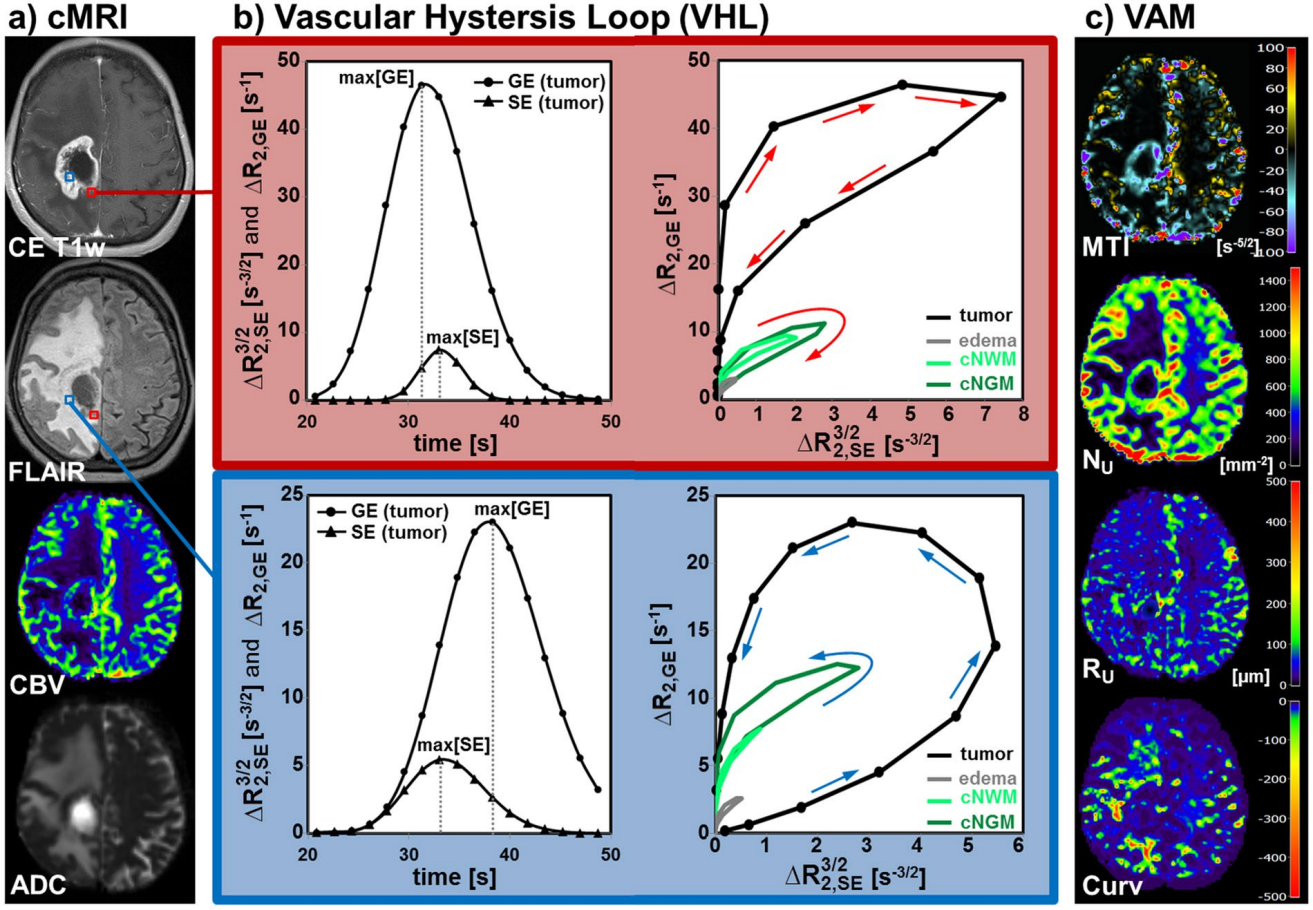
Calculation of vascular hysteresis loops and VAM biomarker maps for a 63-year-old female patient suffering from a recurrent glioblastoma. The presented data were obtained from an examination which was performed before onset of bevacizumab treatment (baseline examination). (**a**) Conventional MRI (cMRI) included contrast-enhanced T1-weighted (CE T1w), FLAIR, GE-EPI DSC perfusion evaluated as maps of cerebral blood flow (CBV), and DWI evaluated as apparent diffusion coefficient (ADC), respectively. (**b**) The ΔR(t)_2,GE_ and ${{\rm{\Delta }}{\rm{R}}(t)}_{2,\text{SE}}^{3/2}\,\,$ curves for the first bolus of the GE- and SE-EPI DSC data, respectively, were fitted to a gamma-variate function (left) and used for calculation of the vascular hysteresis loops (VHLs, i.e. the ${{\rm{\Delta }}{\rm{R}}}_{2,\text{GE}}$ versus ${{\rm{\Delta }}{\rm{R}}}_{2,\text{SE}}^{3/2}$ diagram, right). The red-framed part shows bolus curves and VHLs for a voxel position (red squares in a) with arterial-dominated microvasculature, and the blue-framed the analogue for a voxel position (blue squares in a) with venule/capillary-dominated microvasculature. The arrows indicate the rotational direction of the VHLs. (**c**) The maps of the microvessel type indicator (MTI), the upper limit for microvessel density (N_U_) and radius (R_U_) adapted to the temporal shift phenomenon, and the curvature (Curv) of the VHL (top-down). The color codes are depicted at the right image margins.

Calculation of VHLs and the VAM approach are illustrated in Fig. 1. Interpretation of VHLs was performed in accordance with the findings of Emblem *et al*.^19^ and Xu *et al*.^17^. In areas with faster blood flow velocities, i.e. in voxels with arteriole dominated microvasculature (red squares in Fig. 1a), the GE-EPI perfusion signal peaked earlier than the SE-EPI perfusion signal (left diagram in the red-framed part of Fig. 1b) and the VHL traversed in clockwise direction (right diagram in the red-framed part of Fig. 1b). Whereas, the SE-EPI perfusion signal peaked earlier than the GE-EPI perfusion signal (left diagram in the blue-framed part of Fig. 1b) and the VHL traversed in the counterclockwise direction (right diagram in the blue-framed part of Fig. 1b) if the vascular system in a voxel was dominated by venule- and capillary-like vessel components with slower blood flow velocities (blue squares in Fig. 1a). For purposes of comparison, VHLs for voxel positions with arteriole dominated microvasculature (Fig. 1b, top right) and venule/capillary dominated microvasculature (Fig. 1b, downright) in edema (gray VHL), contralateral normal white matter (cNWM, light green), and contralateral normal gray matter (cNGM, dark green) are depicted in relation to the tumor VHL.

Accordingly to our definition for the calculation of microvessel type indicator (MTI) values, a counterclockwise VHL-direction was associated with a negative signed VHL-area, i.e. a negative MTI, and vice-versa for the clockwise VHL direction. In MTI maps, negative values were assigned to cool colors and positive to warm colors, respectively. Consequently, maps of MTI enabled discrimination between supplying arterial (areas with warm colors in the topmost image in Fig. 1c) and draining venous microvasculature (areas with cool colors in the topmost image in Fig. 1c). Maps of microvessel density (N_U_, second image in Fig. 1c) and microvessel radius (R_U_, third image in Fig. 1c) showed heterogeneous patterns of mildly to severely increased levels. Areas with severely increased N_U_ showed a rather mild increase in R_U_ and vice versa, i.e. maps of N_U_ and R_U_ provided complementary information about the heterogeneity of the microvasculature in GB. The Curv parameter showed almost exclusively negative values. Curv values in the tumor core were similar compared to ipsi- and contralateral normal appearing brain (NAB). Curv was severely decreased, i.e. showed severely larger negative values, in peritumoral vasogenic edema when compared to ipsi- and contralateral NAB.

During bevacizumab treatment, VHLs and VAM biomarker images revealed massive but spatially heterogeneous changes in microvascular architecture. We observed (i) tumor areas with decreased neovascularization activity interpreted as response to AAT; (ii) tumor areas with unchanged and increased neovascularization activity interpreted as non-response to AAT; and (iii) areas with newly developed neovascularization activity interpreted as progression during AAT.

**Figure 2:**
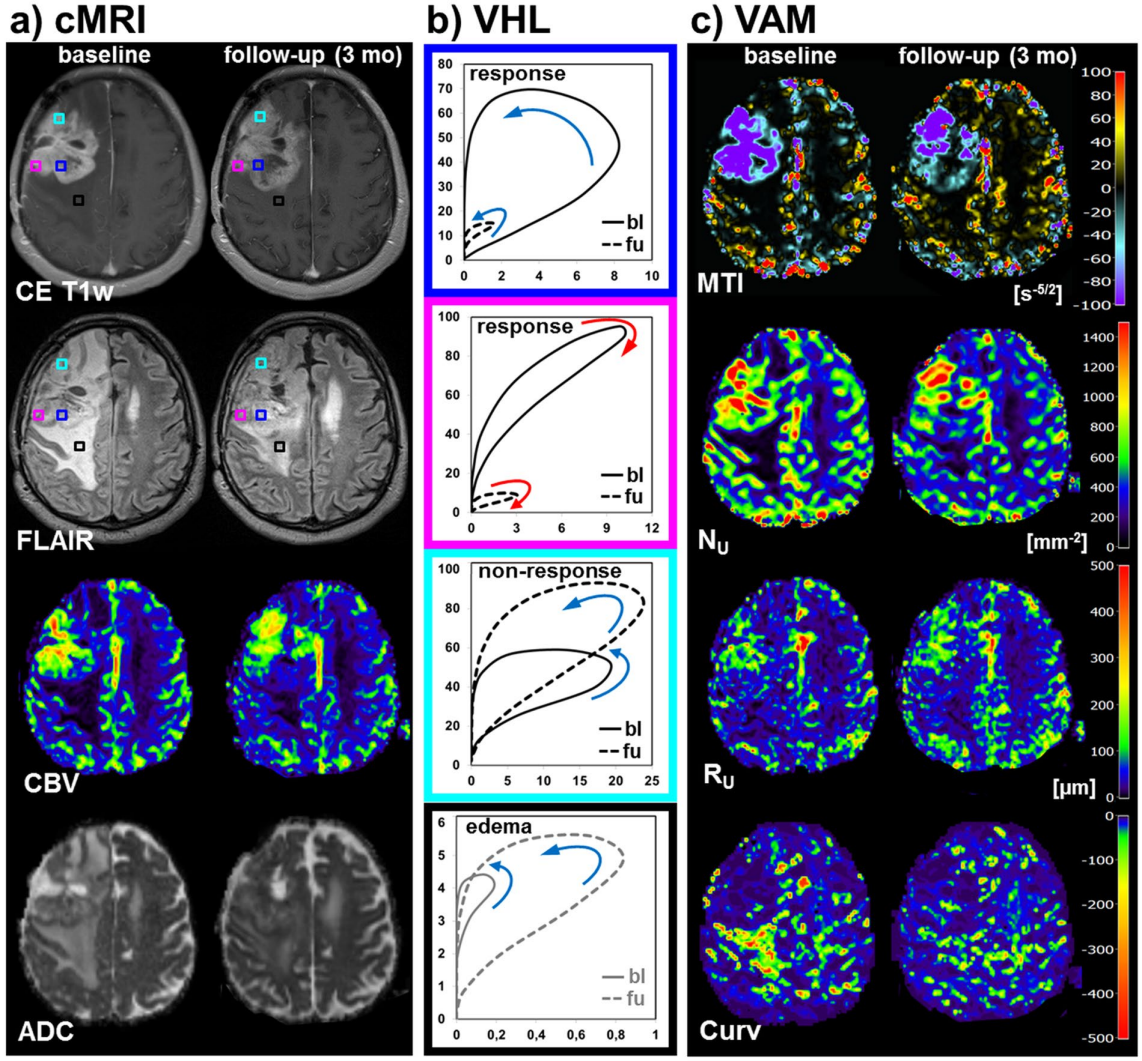
Vascular hysteresis loops and VAM biomarker maps for a 51-year-old male patient suffering from a recurrent glioblastoma before AAT onset (baseline, bl) and at 3-month follow-up (fu). (**a**) Conventional MRI (cMRI) included contrast-enhanced T1-weighted (CE T1w), FLAIR, GE-EPI DSC perfusion evaluated as maps of cerebral blood flow (CBV), and DWI evaluated as apparent diffusion coefficient (ADC), respectively. (**b**) VHLs, i.e. the ${{\rm{\Delta }}{\rm{R}}}_{2,\text{GE}}$ (y-axis) versus ${{\rm{\Delta }}{\rm{R}}}_{2,\text{SE}}^{3/2}$ (x-axis) diagrams, for a voxel position in the responding (blue- and magenta-framed VHL), non-responding tumor (cyan), and for edema in the vicinity of the responding tumor (black). The voxel positions are marked with squares in the CE T1w and FLAIR images in (**a**) and have the same color as the frames of the VHLs. The arrows indicate the rotational direction of the VHLs. (**c**) The maps of the microvessel type indicator (MTI), the upper limit for microvessel density (N_U_) and radius (R_U_) adapted to the temporal shift phenomenon, and the curvature (Curv) of the VHL (top-down). The color codes are depicted at the right image margins.

A representative case of a patient suffering from recurrent GB who showed both response and non-response image parameters to bevacizumab treatment is illustrated in Fig. 2. Compared to the baseline examination response was associated with a distinct reduction in the VHL-area in the follow-up examination. This was observed for both venule/capillary (blue-framed VHLs in Fig. 2b) and arteriole dominated (magenta-framed VHLs in Fig. 2b) microvasculature. Both parameters of the VHL, ${{\rm{\Delta }}{\rm{R}}}_{2,\text{GE}}$ and ${{\rm{\Delta }}{\rm{R}}}_{2,\text{SE}}^{3/2}$, were reduced in the follow-up examination. Interpreting these data means, tumor vasculature was reduced in both larger and smaller microvasculature. The VHLs in non-responding tumor areas showed an increase in area due to both an increase in ${{\rm{\Delta }}{\rm{R}}}_{2,\text{GE}}$ (i.e. larger microvasculature) and ${{\rm{\Delta }}{\rm{R}}}_{2,\text{SE}}^{3/2}$ (i.e. smaller microvasculature; see cyan-framed VHLs in Fig. 2b). Response to antiangiogenic therapy in the posterior tumor part was accompanied with a distinct reduction in peritumoral edema (Fig. 2a) which was associated with an increase in VHL-area (black-framed VHLs in Fig. 2b). As can be seen from the VHLs (black-framed VHLs in Fig. 2b), these vascular changes in peritumoral edema were dominated by an increase in microvascular perfusion (i.e. increase in ${{\rm{\Delta }}{\rm{R}}}_{2,\text{SE}}^{3/2}$). Note: the voxel positions for the VHLs are marked with squares in the CE T1w and FLAIR images in Fig. 2a, which have the same color as the frames of the VHLs. The MTI and N_U_ images clearly revealed a spatial segregation in responding and non-responding tumor areas. The posterior tumor parts showed an increase in MTI (i.e. less negative MTI values) and decrease in N_U_ as indication for decreased neovascularization activity as response to anti-angiogenic treatment. The anterior part of the tumor, however, showed an unaltered high or even increasing neovascularization activity: highly negative or decreasing MTI values, and high or increasing N_U_ values, respectively. The changes in the R_U_ images were less distinct. Reduction in peritumoral edema was accompanied with a massive increase in the Curv parameter (less negative Curv values; see image at the bottom of Fig. 2c).

**Figure 3:**
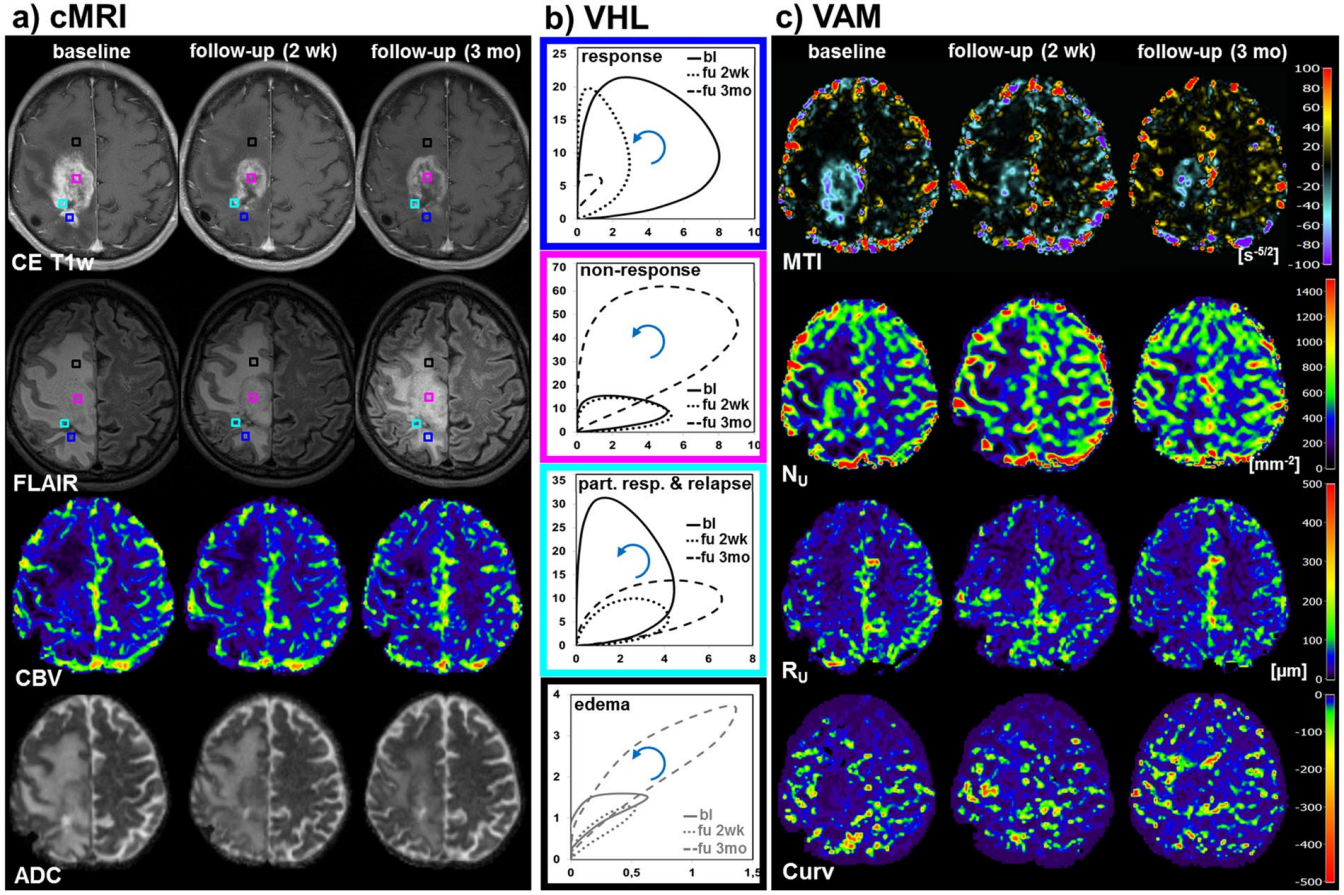
Vascular hysteresis loops and VAM biomarker maps for a 54-year-old male patient suffering from a recurrent glioblastoma before AAT onset (baseline, bl), at early 2-week follow-up (“fu 2wk”), and at 3-month follow-up (“fu 3mo”). (**a**) Conventional MRI (cMRI) included contrast-enhanced T1-weighted (CE T1w), FLAIR, GE-EPI DSC perfusion evaluated as maps of cerebral blood flow (CBV), and DWI evaluated as apparent diffusion coefficient (ADC), respectively. (**b**) VHLs, i.e. the ${{\rm{\Delta }}{\rm{R}}}_{2,\text{GE}}$ (y-axis) versus ${{\rm{\Delta }}{\rm{R}}}_{2,\text{SE}}^{3/2}$ (x-axis) diagrams, for a voxel position in the responding (blue-framed VHLs), non-responding (magenta), partial responding tumor with relapse (cyan), and for edema in the vicinity of the non-responding tumor (black). The voxel positions are marked with squares in the CE T1w and FLAIR images in (**a**) and have the same color as the frames of the VHLs. The arrows indicate the rotational direction of the VHLs. (**c**) The maps of the microvessel type indicator (MTI), the upper limit for microvessel density (N_U_) and radius (R_U_) adapted to the temporal shift phenomenon, and the curvature (Curv) of the VHL (top-down). The color codes are depicted at the right image margins.

Figure 3 illustrates a patient suffering from a recurrent GB who obtained an additional MR examination including the VAM experiments for early AAT response detection after the first dose of bevacizumab, i.e. after 2 weeks. The second follow-up examination was performed after 3 months of AAT onset, as with all the other patients. In this patient, responding tumor vasculature showed very similar changes between the baseline and the 3-month follow-up examination compared with those in Fig. 2 (compare blue-framed VHLs in Figs 2b and 3b): ${{\rm{\Delta }}{\rm{R}}}_{2,\text{GE}}$ and ${{\rm{\Delta }}{\rm{R}}}_{2,\text{SE}}^{3/2}$, were reduced in the 3-month follow-up examination. Interestingly, the VHL at the early follow-up after 2 weeks showed only a reduction in ${{\rm{\Delta }}{\rm{R}}}_{2,\text{SE}}^{3/2}$, i.e. a therapeutic effect in the small microvasculature of the tumor (blue-framed VHLs in Fig. 3b). Non-responding tumor vasculature showed also similar changes between the baseline and the 3-month follow-up examination compared with those in Fig. 2 (compare magenta-framed VHLs in Figs 2b and 3b): ${{\rm{\Delta }}{\rm{R}}}_{2,\text{GE}}$ and ${{\rm{\Delta }}{\rm{R}}}_{2,\text{SE}}^{3/2}$, were increased in the 3-month follow-up examination. In the 2-week follow-up, however, no change occurred in the VHL compared to baseline (magenta-framed VHLs in Fig. 3b). In this patient we additionally detect an area with partial response after the first dose bevacizumab (2-week follow-up) characterized with a strong reduction in ${{\rm{\Delta }}{\rm{R}}}_{2,\text{GE}}$ but not in ${{\rm{\Delta }}{\rm{R}}}_{2,\text{SE}}^{3/2}$, followed by a relapse at the 3-month follow-up characterized by an increase in both ${{\rm{\Delta }}{\rm{R}}}_{2,\text{GE}}$ and especially in ${{\rm{\Delta }}{\rm{R}}}_{2,\text{SE}}^{3/2}$ (cyan-framed VHLs in Fig. 3b). Again, the MTI and N_U_ images clearly revealed a spatial segregation in responding, non-responding, partial responding and relapsing tumor areas (Fig. 3c). Although the tumor showed areas with non-response, partial response, relapse, and progression, FLAIR images showed a reduction in peritumoral edema accompanied by “normalization” of the VHL at the selected voxel position. The Curv image, however, revealed dynamic changes in the whole peritumoral area (image at the bottom of Fig. 3c **)**.

**Figure 4:**
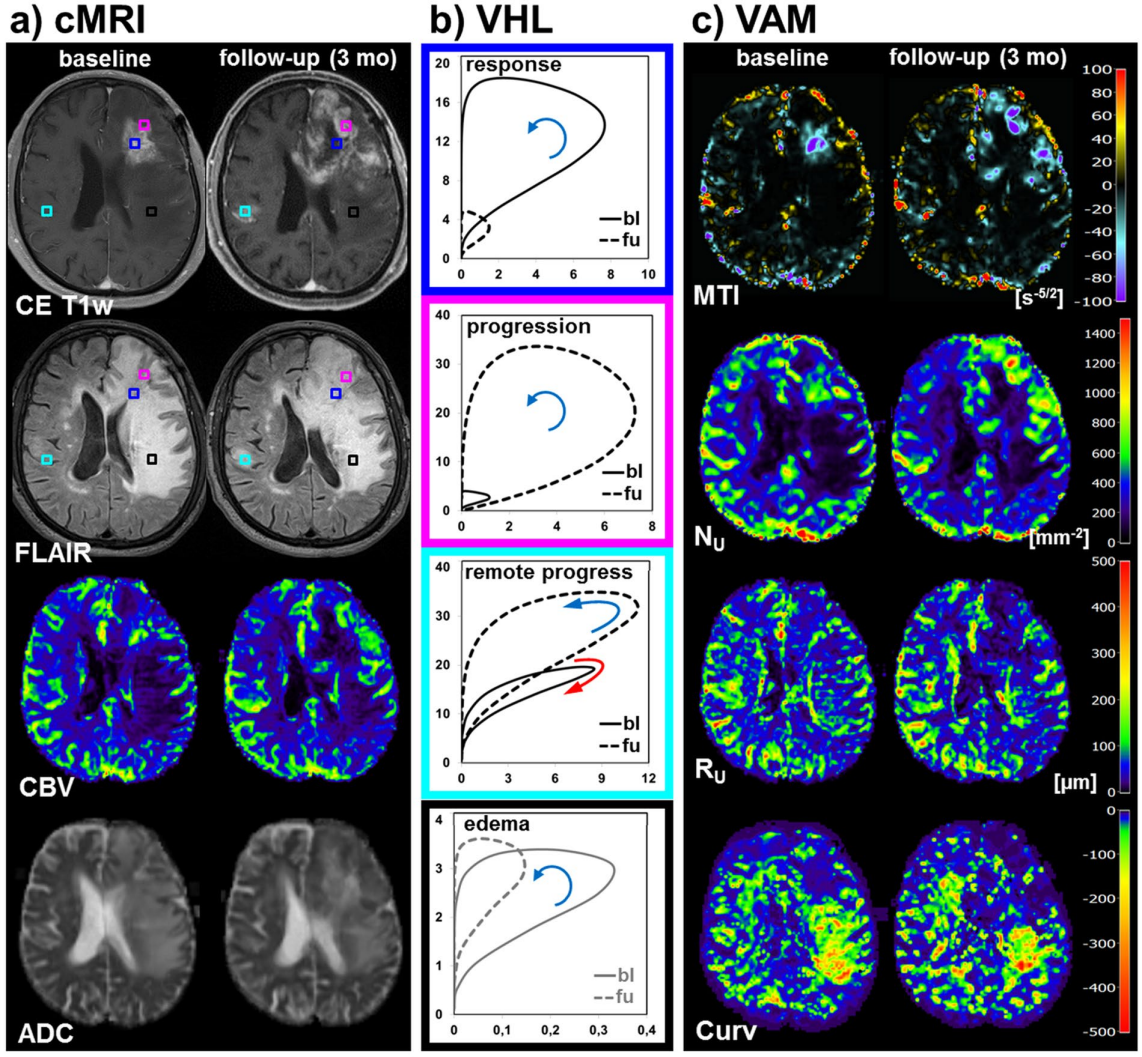
Vascular hysteresis loops and VAM biomarker maps for a 76-year-old male patient suffering from a recurrent glioblastoma before AAT onset (baseline, bl) and at 3-month follow-up (fu). (**a**) Conventional MRI (cMRI) included contrast-enhanced T1-weighted (CE T1w), FLAIR, GE-EPI DSC perfusion evaluated as maps of cerebral blood flow (CBV), and DWI evaluated as apparent diffusion coefficient (ADC), respectively. (**b**) VHLs, i.e. the ${{\rm{\Delta }}{\rm{R}}}_{2,\text{GE}}$ (y-axis) versus ${{\rm{\Delta }}{\rm{R}}}_{2,\text{SE}}^{3/2}$ (x-axis) diagrams, for a voxel position in the responding tumor (blue-framed VHLs), tumor progression (magenta), remote progression (cyan), and for edema in the vicinity of the tumor progression (black). The voxel positions are marked with squares in the CE T1w and FLAIR images in (**a**) and have the same color as the frames of the VHLs. The arrows indicate the rotational direction of the VHLs. (**c**) The maps of the microvessel type indicator (MTI), the upper limit for microvessel density (N_U_) and radius (R_U_) adapted to the temporal shift phenomenon, and the curvature (Curv) of the VHL (top-down). The color codes are depicted at the right image margins.

A patient with response, progression, and remote progression in the contralateral hemisphere during AAT is depicted in Fig. 4. Again, the changes in the VHLs for the responding tumor vasculature were similar compared to the other patients (compare blue-framed VHLs in Figs 2b, 3b and 4b). This patient, however, showed progression of the GB almost into the entire vicinity. A representative VHL in this area showed an increase in both ${{\rm{\Delta }}{\rm{R}}}_{2,\text{GE}}$ and ${{\rm{\Delta }}{\rm{R}}}_{2,\text{SE}}^{3/2}$ due to the tumor progression (magenta-framed VHLs in Fig. 4b). A small area in the remote progression in the contralateral hemisphere showed even a change from an arterial-dominated to a venule/capillary-dominated vasculature (cyan-framed VHLs in Fig. 4b). These distinct changes in neovascularization activity were clearly detected and visualized in the MTI and N_U_ maps, respectively. Additionally, the posterior part of the edema showed no regression but partly even a progression on the Curv map (image at the bottom of Fig. 4c **)**, which is accompanied with a further reduction in both ${{\rm{\Delta }}{\rm{R}}}_{2,\text{SE}}^{3/2}$ and VHL-area (black-framed VHLs in Fig. 4b).

**Figure 5:**
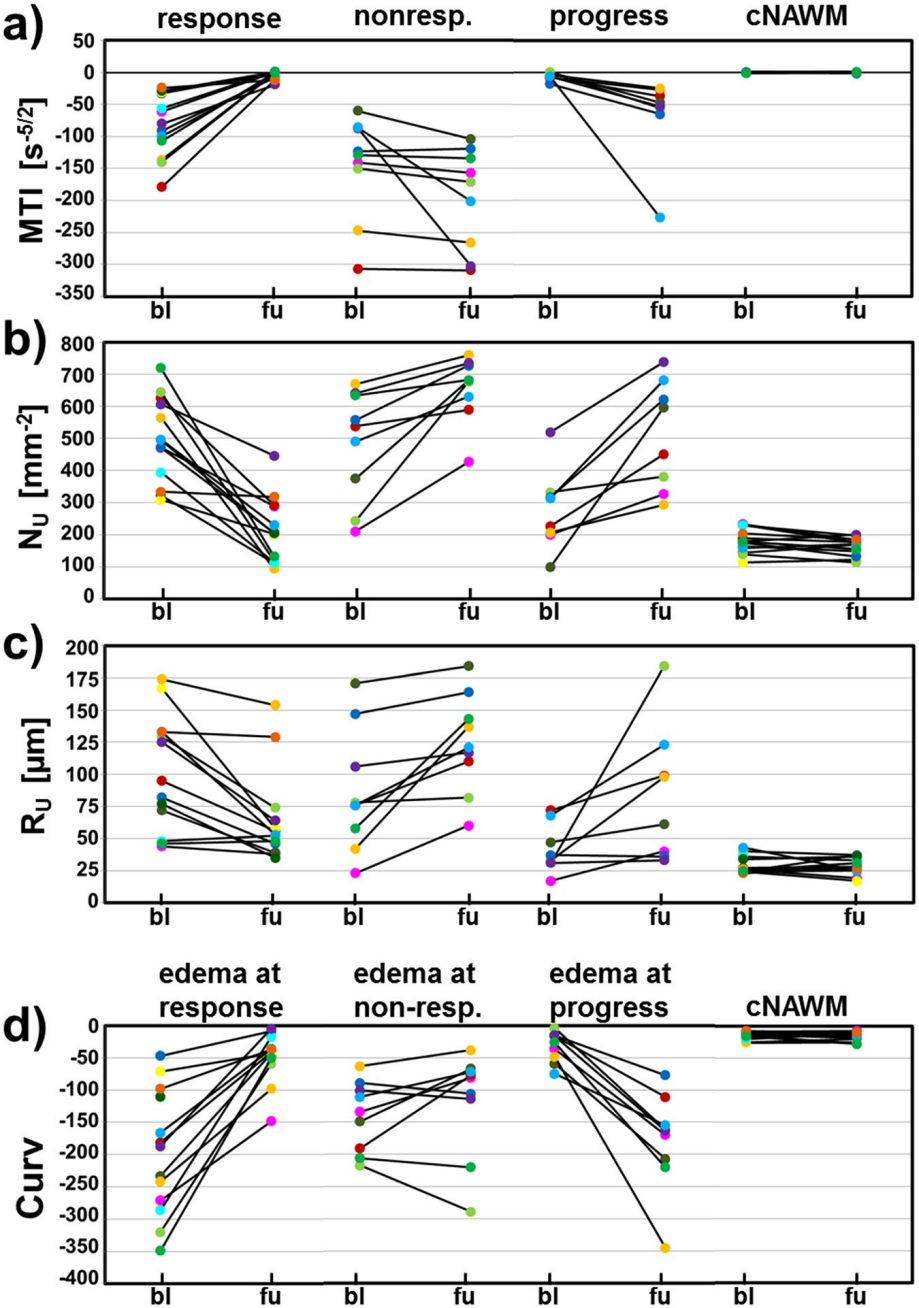
Changes for the (**a**) MTI, (**b**) N_U_, and (**c**) R_U_ for each patient individually in responding (all 13 patients), non-responding (9 patients) tumor, and in tumor progression (8 patients). (**d**) Shows the changes of the Curv parameter in peritumoral edema associated with the tumor areas in (**a**–**c**): edema in the vicinity of responding tumor, non-responding tumor, and tumor progression, respectively. Note: bl = baseline; fu = 3-month follow-up.

All 13 patients included in this study showed areas with response to bevacizumab, nine patients additionally showed non-response, and eight patients additionally showed a progression of the GB during AAT. The changes for the MTI, N_U_, and R_U_ in these three different tumor areas for each patient individually are depicted in Fig. 5a–c. The changes of the Curv parameter in peritumoral edema in the vicinity of responding, non-responding, and progressive tumor parts are depicted in Fig. 5d. Mean values (±sd) for the four VAM biomarkers in the three different tumor areas and the associated peritumoral edematous areas are summarized in Table 1. MTI, N_U_, and R_U_ were significant different between the 3-month follow-up compared to base-line: responding tumor areas showed an increase in MTI and a decrease in N_U_ and R_U_; non-responding tumor areas and progression, however, showed a decrease in MTI and an increase in N_U_ and R_U_. As realized from investigation of the individual cases, Curv was most sensitive to changes in peritumoral edema.

**Table 1:** VAM biomarker values in three different tumor areas and peritumoral edema.

	Ex.	Tumor response	Tumor non-resp.	Tumor progress	Edema response	Edema non-resp.	Edema progress	cNAB
		(N = 13)^a^	(N = 9)^a^	(N = 8)^a^	(N = 13)^a^	(N = 9)^a^	(N = 8)^a^	(N = 13)^a^
MTI	bl	−82.2 ± 60.4	−147.8 ± 80.2	−6.0 ± 5.8	0.3 ± 1.0	−0.4 ± 1.1	−0.5 ± 1.2	0.0 ± 0.7
[s^−5/2^]	fu	−4.2 ± 6.4*	−196.0 ± 78.7*	−67.1 ± 66.1*	−1.4 ± 2.7	−0.1 ± 2.6	0.4 ± 2.9	0.2 ± 0.6
N_U_	bl	498 ± 132	484 ± 171	277 ± 125	101 ± 52	120 ± 43	151 ± 53	180 ± 37
[mm^−2^]	fu	212 ± 103*	656 ± 101*	511 ± 170*	173 ± 94*	162 ± 43*	129 ± 48	164 ± 28
R_U_	bl	102 ± 45	86 ± 48	42 ± 19	83 ± 44	61 ± 18	43 ± 19	29 ± 7
[µm]	fu	65 ± 36*	124 ± 39*	84 ± 53*	54 ± 27	77 ± 33	91 ± 28*	28 ± 6
Curv	bl	−7 ± 6	−8 ± 11	−22 ± 22	−197 ± 97	−140 ± 55	−33 ± 24	−15 ± 6
	fu	−85 ± 103*	−30 ± 79	−11 ± 13	−49 ± 7*	−118 ± 82	−179 ± 76*	−17 ± 8

Values are mean values ± standard deviation.

^a^In parentheses: number of patients.

*Values marked with an asterisks are significant (P < 0.05) different to the value at the baseline examination.

Note: MTI = microvessel type indicator; N_U_ = microvessel density; R_U_ = microvessel radius; Curv = curvature of the long-axis of the VHL; Exam. = examination; bl = baseline; fu = 3-month follow-up, cNAB = contralateral normal appearing brain.

Comparison of VAM biomarker values at baseline examination between tumor regions which showed response and non-response at the 3-month follow-up in order to check for potential predicting factors revealed significant (*P* = 0.042) higher MTI values at baseline in tumor areas which responded in the follow-up (MTI(baseline)_response_ = −82.2 ± 60.4) compared to the tumor area which not responded in the follow-up (MTI(baseline)_non-response_ = −147.8 ± 80.2). All the other VAM biomarkers showed no significant differences at baseline.

## Discussion

Analysis of VHLs in combination with the VAM approach demonstrated three features for investigation of the microvasculature of GBs: **i**) estimation of the microvascular compartments (ratio of larger vs. smaller microvessel); **ii)** estimation of the microvascular type (arterioles vs. capillaries vs. venules); and **iii)** estimation of the spatial heterogeneity of neovascularization activity and its changes during AAT by evaluating features **i** and **ii** via calculation of VAM biomarker images.

Firstly, investigation of the two VHL-variables, especially of the maxima of ${{\rm{\Delta }}{\rm{R}}}_{2,\text{GE}}$ and ${{\rm{\Delta }}{\rm{R}}}_{2,\text{SE}}^{3/2}$, allowing for a vessel-caliber-dependent estimation of the microvascular compartments within a voxel. A voxel dominated by larger microvessels showed relatively higher max[${{\rm{\Delta }}{\rm{R}}}_{2,\text{GE}}$] and vice-versa for a voxel dominated by smaller microvessels with higher max[${{\rm{\Delta }}{\rm{R}}}_{2,\text{SE}}^{3/2}$]. This feature enabled us to differentiate the effects of bevacizumab on larger and smaller microvasculature: early 2-week follow-up showed a reduction in max[${{\rm{\Delta }}{\rm{R}}}_{2,\text{SE}}^{3/2}$] (i.e. smaller microvasculature) in responding tumor regions, but no or minor changes in max [${{\rm{\Delta }}{\rm{R}}}_{2,\text{GE}}$]. In the 3-month follow-up, however, responding tumor regions showed a reduction in max [${{\rm{\Delta }}{\rm{R}}}_{2,\text{GE}}$] (larger microvasculature) too. To the best of our knowledge this differentiation of effects of bevacizumab on the larger and smaller microvasculature at early and delayed follow-up was not described so far. Emblem *et al*.^19^ recently built on the hysteresis effect, which was originally described by Kiselev *et al*.^13^ more than a decade ago, and demonstrated that AAT of recurrent GBs using cediranib can improve microcirculation and reduce vessel calibers. This response was associated with prolonged patient survival. An analysis of the dynamic changes of the VHLs, however, was not performed in this study.

Secondly, analysis of the VHL-direction enables the estimation of the microvascular type which dominates a voxel: faster-inflow (arterioles-dominated) vs. slow-inflow (venules-dominated) assessed by rotational direction of the VHL (clockwise vs. counterclockwise). This was previously described with computer simulations under consideration of a tree model of microvasculature^17^ and was expanded by the temporal shift phenomenon between max[${{\rm{\Delta }}{\rm{R}}}_{2,\text{SE}}^{3/2}]$ and ${\max [{\rm{\Delta }}{\rm{R}}}_{2,\text{GE}}]$ ^19^. A detailed interpretation of the temporal shift, however, was not presented so far. Considering both the maxima and the direction of the VHL may be helpful to explain the temporal shift phenomenon. In a voxel dominated by larger (faster-flow) arterioles and capillaries the blood with the first-pass contrast agent bolus flows from arterioles with lager diameter (GE signal peak) followed by capillaries with smaller diameter (SE signal peak), i.e. the GE signal rises and peaks before the SE signal resulting in a clockwise rotating VHL and a separation of maxima. The same is true for a voxel dominated by (slower-flow) venules and capillaries: the contrast agent bolus flows from capillaries to venules and the SE signal rises and peaks before the GE signal. The temporal shift leads to a VHL with two or more reversal points between max[${{\rm{\Delta }}{\rm{R}}}_{2,\text{SE}}^{3/2}]$ and ${\max [{\rm{\Delta }}{\rm{R}}}_{2,\text{GE}}]$. Furthermore, the temporal shift is the stronger, the longer the transit times through the capillary bed, the larger the capillary bed volume, and the more inefficient the capillary bed microstructure. A temporal shift between max[${{\rm{\Delta }}{\rm{R}}}_{2,\text{SE}}^{3/2}]$ and ${\max [{\rm{\Delta }}{\rm{R}}}_{2,\text{GE}}]$ of more than one TR of the DSC sequences (i.e. 1.74 s in this study) is associated with more than two reversal points. In other words, the VHL segment of reversal represents the capillary compartment. This is in accordance with results from computer simulations^17^. It is well-known that neovascularization in GBs is highly defective, resulting in a disorganized, abundant and aberrant vasculature with tortuous vessels^2^ and abnormal capillary bed topology^3^. This causes heterogeneity in blood flow and perfusion through the tumor vasculature^20^. Interestingly, VHLs with a temporal shift has already been shown in the previous studies from Kiselev *et al*.^13^ (see Fig. 5b) and Xu *et al*.^17^ (see Fig. 2b).

Finally, the VAM approach allowing for investigation of the spatial heterogeneity of neovascularization activity and microvascular changes due to bevacizumab treatment by evaluating the first two features via calculation of biomarker images. Maps of the VAM biomarker which were adapted to the temporal shift phenomenon provided a complementary insight into the heterogeneous structure of microvasculature pathologies in glioma as consequence to angiogenetic activities initiated by the lesions. Furthermore, we detected changes in VAM biomarker images which can be interpreted as microvascular response, non-response, and progression during AAT. Bevacizumab-untreated tumor regions (at baseline) with lower neovascularization activity (less negative MTI) tended rather to show response to AAT in the follow-up than regions with higher neovascularization activity. This might be an indication for the known vascular niche which is thought of as protective microenvironment that enables glioblastoma stem cells to freely proliferate and remain undifferentiated, completely unaffected by any external influences^21, 22^. The Curv parameter might be a marker for the severity of vasogenic edema and compression of small microvessels due to increased interstitial pressure in the edema. However, there is no verified histopathologic basis for an interrelationship so far. Correlations with DTI data, histologic parameters, and Monte Carlo simulations for VHL formation could be informative.

This study has several limitations. First of all, the dual contrast agent injections approach in combination with two separate SE- and GE-DSC acquisitions is significantly more sensitive to both patient motions and variations in injection timing compared to a combined GE-SE sequence. Our strategies help to minimize the probability of these sources of error but cannot eliminate them entirely. Moreover, the injection of a double dose of contrast agent with two separate injections is required. On the other hand, our approach with separate SE- and GE-DSC sequences allowing the acquisition of VAM data with high SNR, high spatial resolution, and coverage of the whole brain, which is mandatory for routine GE-EPI perfusion. The combined GE-SE sequence used in previous studies^19, 23–25^, however, does not meet these requirements and may necessitate performing the routine GE perfusion in addition or in a separate study, which consequently requires a further contrast agent injection. This might be a reason for the limited application in clinical routine of this promising technique. Additionally, most previous studies published used a double-dose for the combined GE-SE perfusion^13, 19, 24, 25^, and/or applied a second injection for perfusion MRI^19, 23, 25^ or correction of leakage effects^24^. The efforts to upgrade the combined GE-SE perfusion sequence with the promising Simultaneous-MultiSlice (SMS) technique^26^ for full coverage may help to overcome these limitations. The interpolation to the target resolution, which potentially influences the magnitude of the measured susceptibility effect, and the short TE, which reduces contrast agent sensitivity and hence SNR, of the SE-EPI perfusion sequence are a further limitations. Applying a larger acquisition matrix would increase TR and consequently the temporal resolution. This is also true for a longer TE. The accuracy of the hysteresis quantification, however, is critically dependent on a high temporal resolution.

In summary, analysis of VHLs in combination with VAM biomarkers is capable of both, assessing the topological and structural heterogeneity of tumor microcirculation, and monitoring the response to and the progression during bevacizumab treatment. This approach may lead to a new perspective on investigating microvascular changes in glioma and other brain pathologies as well as on therapy monitoring. However, validations of the biomarkers are required to enable rational interpretation.

## Methods

### Patients

This study was approved by the Institutional Review Boards of the University of Erlangen and the University Clinic of St. Pölten. Analysis of patient data and all methods were performed in accordance with both the Declaration of Helsinki and the relevant guidelines and regulations. Written informed consent for both study participation and publication of information/images was obtained from all subjects. Between July 2015 and December 2016, fifteen patients (7 women, 8 men; 55.6 ± 13.3 years) suffering from recurrent glioblastoma who received bevacizumab (Avastin®, Roche; every 2 weeks 10 mg/kg-bodyweight) as second-line mono-therapy were included in this study. From the 15 patients, two patients (13%) with severe neurological deficits were excluded due to patient motion between or during the MR scans.

### MR Imaging

All MRI studies were performed on a 3 Tesla clinical MR scanner (Tim Trio; Siemens, Erlangen, Germany) equipped with a standard 12-channel head coil. MR examinations were performed 1–5 days prior to (baseline) and 3 months after bevacizumab treatment onset (follow-up). In two patients a MRI examination was additionally performed after the first cycle of bevacizumab (two weeks after onset) in order to check for early response to treatment.

The clinical routine MRI protocol included an axial fluid-attenuated inversion-recovery (FLAIR) sequence (TR/TE/TI: 5000/460/1800 ms; in-plane resolution: 0.45 × 0.45 mm, slice thickness: 3 mm; 48 slices; 2 averages; acquisition time, TA: 3.85 min), a single-shot diffusion-weighted echo-planar imaging (DW-EPI) sequence (TR/TE: 5300/98 ms; in-plane resolution: 1.2 × 1.2 mm, slice thickness: 4 mm; 29 slices; 4 averages; parallel imaging using generalized autocalibrating partially parallel acquisition (GRAPPA)^27^ factor of 2, b-values of 0 and 1000 s/mm^2^; TA: 1.3 min), and pre-and post-contrast enhanced (CE) T1-weighted GE sequences (TR/TE: 250/2.8 ms; in-plane resolution: 0.5 × 0.5 mm, slice thickness: 4 mm; 29 slices; 2 averages).

For VAM we used a dual contrast agent injections approach in order to obtain DSC perfusion MRI data using SE- and GE-EPI sequences with high signal-to-noise ratio (SNR), high spatial resolution, and coverage of the whole brain. However, patient motions between the two scans as well as differences in the time to first-pass peak may significantly affect the data evaluation^18^. To minimize the probability of these sources of error we used the following strategies as described previously^28^:

**i**) To prevent patient motion, special caution was made to properly fix the head and provide clear and repeated patient instructions before and during the MRI examination. Due to the fact that patient motions during or between the SE- and GE-EPI DSC perfusion scans may significantly affect a pixel-based analysis, we used the approach described by Hsu *et al*.^18^ to detect patient motion: During data preprocessing, dynamic masks for the contours from the SE- and GE-EPI perfusion raw data were created. By matching the contours, we checked for patient motion between and during the scans^18^. A mismatch of more than one pixel was defined as ‘severe patient motion’, which was an exclusion criterion of this study from further evaluation. This was necessary in two patients as mentioned above. For the remaining 13 patients we did not observe significant motions, i.e. contour mismatch was not more than one pixel, and thus no image coregistration was applied during data analysis.

**ii)** To prevent time differences of the first-pass peak signal between the contrast agent boluses a peripheral pulse unit (PPU) was fitted to a finger of the patients in order to monitor heart rate and cardiac cycle. Special attention was paid to perform the two injections at the same heart rate and exactly at the same phase of the cardiac cycle (at PPU’s peak systole signal). Due to the fact that timing differences between the SE- and GE-EPI DSC perfusion scans may significantly affect a pixel-based analysis, we additionally checked for any error in injection timing. During data preprocessing the arterial input functions (AIFs) for the SE- and GE-EPI DSC perfusion data were verified for correct timing, i.e. were checked for differences in the time to peak (TTP) between the two AIFs. For all 28 examinations included in this study we did not observe differences between the SE- and GE-AIFs of the respective examination.

Geometric parameters (section orientation, angulation, field-of-view, etc.) and measurement parameters were chosen identical for the SE- and GE-EPI DSC sequences: TR, 1740 ms; in-plane resolution, 1.8 × 1.8 mm; SE-EPI were spatially interpolated to a 128 × 128 matrix; slice thickness, 4 mm; 29 slices; GRAPPA, 2; 60 dynamic measurements; and TA: 2.0 min, respectively. TE was different between SE- (33 ms) and GE-EPI (22 ms). Both DSC perfusion MRI examinations were performed with administration of 0.1 mmol/kg-bodyweight gadoterate meglumine (Dotarem, Guerbet) at a rate of 4 ml/s using a MR-compatible injector (Spectris, Medrad) and with utmost caution for the injection time (see above). A 20 ml bolus of saline was injected subsequently at the same rate. The first DSC MRI was obtained by the SE-EPI technique since SE-EPI DSC perfusion MRI is less sensitive to contrast-agent leakage^29, 30^. CE T1-weighted images in axial, sagittal, and coronal orientation were performed between two DSC perfusion acquisitions. The total acquisition time of the three CE T1w sequences was 8 minutes. Thereafter the second DSC MRI was obtained using the GE-EPI technique during a bolus injection under the same conditions as for the SE-EPI DSC acquisition.

### MRI data processing

VAM analysis was performed using custom-made in-house software developed using MATLAB (MathWorks, Natick, MA, USA). SE- and GE-EPI DSC data were imported into the software and checked for motion artifacts during or between the acquisitions^18^. Next, AIFs for the SE- and GE-EPI DSC data were automatically identified in each slice using automatic segmentation of brain tissue sub-classes in DSC concentration-time curves. In short, concentration-time curves were assumed to belong to one of five tissue sub-classes (white matter, gray matter, arterial blood, venous blood, or ‘other’), and multiple features (first moment of the concentration–time curves integral, peak height, time to peak height, and bolus arrival time) were used to assign each concentration–time curves into the most probable tissue class. The average of five concentration-time curves with the lowest first moment and the highest average peak height was then selected as the final AIF^31^. ΔR(t)_2,SE_ and ΔR(t)_2,GE_ were calculated from the signals of the SE- and GE-EPI DSC data using the following equation:

1 $${{\rm{\Delta }}{\rm{R}}(t)}_{2,\text{XE}}=\,-\frac{1}{{{\rm{TE}}}_{{\rm{XE}}}}\cdot \,\mathrm{ln}\,\frac{{S(t)}_{{\rm{XE}}}}{{{\rm{S}}}_{0,\text{XE}}}$$

where XE stands for SE or GE, respectively. TE is the echo time, S_0_ is the baseline (prebolus) signal, and S(t) is the signal during the first bolus passage of the corresponding sequence. S_0_ was determined as the mean of the signals from the 4^th^ to the 15^th^ dynamic volume^18^. For S(t), the respective DSC perfusion MR data were corrected for remaining contrast agent extravasation^32, 33^ and a minimum search between the first and the second bolus passage (caused by recirculation) was performed as described previously^17^ in order to separate the boluses. The truncated ΔR(t)_2,SE_ and ΔR(t)_2,GE_ curves of the first bolus were fitted to a previously described gamma-variate function^34^ and used for calculation of the ${{\rm{\Delta }}{\rm{R}}}_{2,\text{GE}}$ versus ${{\rm{\Delta }}{\rm{R}}}_{2,\text{SE}}^{3/2}$ diagram (Fig. 1b), which we termed vascular hysteresis loop (VHL).

### MR imaging biomarkers of microvascular architecture

The VHL of each voxel was evaluated by the following four parameters: **i)** microvessel type indicator (MTI) as the signed area of the VHL; **ii)** microvessel density (N_U_) and **iii)** radius (R_U_) which were adapted of the temporal shift phenomenon; and **iv)** the curvature (Curv) of the long-axis of the VHL (Fig. 1c). We termed this approach vascular architecture mapping (VAM).

In a first step, the MTI parameter was defined as the area of the VHL signed with the rotational direction of the VHL, i.e. clockwise VHL-direction was identified with a plus-sign and counterclockwise VHL-direction was identified with a minus-sign. MTI values were calculated by subtraction of the areas under the ascending branch of the VHL minus the descending branch of the VHL (we refer to the VHLs for tumor in Fig. 1b). The maximum ${{\rm{\Delta }}{\rm{R}}}_{2,\text{SE}}^{3/2}$ value was defined as reversal point.

In the second step, the temporal shift phenomenon between the SE- and GE-EPI DSC signal time courses was considered by calculation of the maximum Q-index

2 $${{\rm{Q}}}_{{\rm{\max }}}=\,{\rm{\max }}[{{\rm{\Delta }}{\rm{R}}}_{2,\text{GE}}]/\,{\rm{\max }}[{{\rm{\Delta }}{\rm{R}}}_{2,\text{SE}}^{3/2}]$$

for each VHL. This is equivalent to the calculation of the slope of the diagonal of the enveloping rectangle of the VHL. Q_max_ takes into account that the maxima of the ${{\rm{\Delta }}{\rm{R}}}_{2,\text{GE}}$ and ${{\rm{\Delta }}{\rm{R}}}_{2,\text{SE}}^{3/2}$ values, i.e. max[${{\rm{\Delta }}{\rm{R}}}_{2,\text{GE}}$] and max[${{\rm{\Delta }}{\rm{R}}}_{2,\text{SE}}^{3/2}$], are separated in the VHL when a vascular-architecture-induced temporal shift between the dynamic SE- and GE-perfusion MRI signals exists (see VHLs for tumor in Fig. 1b).

Thirdly, N_U_ and R_U_, which previously introduced by Jensen *et al*.^35^, were adapted to the temporal shift phenomenon by using the following equations

3 $${{\rm{N}}}_{{\rm{U}}}=\frac{{{\rm{Q}}}_{{\rm{\max }}}}{{\rm{b}}}\cdot {(\frac{{{\rm{CBV}}}^{2}}{4{{\rm{\pi }}}^{2}\cdot \text{ADC}\cdot {\bar{{\rm{R}}}}^{4}})}^{1/3}$$

and

4 $${{\rm{R}}}_{{\rm{U}}}={(\frac{{\rm{CBV}}\cdot \text{ADC}\cdot {{\rm{b}}}^{3}}{2{\rm{\pi }}\cdot {{\rm{Q}}}_{{\rm{\max }}}^{3}})}^{1/2}$$

where ADC is the apparent diffusion coefficient which was calculated from the DW-EPI MRI data. CBV is the cerebral blood volume which was calculated from the GE-EPI DSC perfusion MRI data. $\bar{{\rm{R}}}$ is the mean vessel lumen radius ($\bar{{\rm{R}}}$ ≈ 3.0 μm) and b is a numerical constant (b = 1.6781) as described previously^35^, which has nothing to do with diffusion weighting.

Finally, the Curv parameter was introduced to assess the shape of the VHL deviating from a symmetric ellipse which is associated with differences in the width of the GE- and SE-EPI perfusion boluses. The Curv values were calculated by fitting of the long-axis of the VHL with a quadratic polynomial followed by determination of the second derivative.

### Statistics

Software (SPSS 14, IBM, Chicago, IL, USA) was used for statistical evaluation. The mean values for the four VAM biomarkers within seven regions of interest (ROI) were calculated. These ROIs were manually defined by an experienced neuroradiologist (A.D.) based on features seen in FLAIR images, CE T1w MRIs, and VAM images, respectively. ROIs were located in: **i)** tumor regions responding to bevacizumab with decreased neovascularization; **ii)** non-responding tumor regions with unchanged or increased neovascularization; **iii)** areas with newly developed neovascularization activity interpreted as progression during AAT; edema in the vicinity of **iv)** responding; **v)** non-responding; and **vii)** progressive tumor **vii)** as well as contralateral normal appearing brain tissue (cNAB) of predominantly white matter. Differences in VAM biomarkers between baseline and 3-month follow-up examination were determined using a Wilcoxon signed-rank test. In order to check for potential predicting factors, we compared VAM biomarker values at baseline examination between tumor regions which showed response and non-response at the 3-month follow-up, respectively, using a Mann-Whitney *U* test. *P* values of less than 0.05 were considered to indicate significance.
